# Effects of a Hypertension Management Mobile App on Urinary Sodium Excretion in Patients With Chronic Kidney Disease: Randomized Controlled Trial

**DOI:** 10.2196/68447

**Published:** 2026-03-16

**Authors:** Takayuki Kawaoka, Yusuke Sakaguchi, Tatsufumi Oka, Yohei Doi, Ryohei Yamamoto, Isao Matsui, Masayuki Mizui, Jun-Ya Kaimori, Yoshitaka Isaka

**Affiliations:** 1 Department of Nephrology, Graduate School of Medicine, The University of Osaka, 2-2-D11, Yamada-oka, Suita, 565-0871, Japan, 81 6-6879-3230; 2 Health and Counseling Center, The University of Osaka, Toyonaka, Japan

**Keywords:** smartphone app–based intervention, salt restriction, chronic kidney disease, hypertension, lifestyle modification

## Abstract

**Background:**

Excessive salt intake is detrimental to the kidneys. Nevertheless, salt restriction is often suboptimal in patients with chronic kidney disease (CKD). Smartphone app–based interventions might help reduce salt intake by supporting self-monitoring and behavior change at scale. However, clinical trials evaluating these interventions for salt reduction are limited, particularly in CKD populations.

**Objective:**

This study investigated whether a hypertension management app could reduce urinary sodium excretion in patients with CKD.

**Methods:**

This open-label, single-center, randomized clinical trial included 101 patients with CKD who had a history of hypertension and estimated 24-hour urinary sodium excretion of 100 mmol or greater. Patients in the intervention group used CureApp HT, a smartphone app designed to manage hypertension through lifestyle modifications and self-monitoring, particularly for salt restriction. The app delivered daily, individualized guidance tailored to each patient’s lifestyle. Patients also received lifestyle counseling by nephrologists during outpatient visits. The control group received lifestyle counseling alone. The intervention period was 12 weeks. The primary outcome was the change in estimated 24-hour urinary sodium excretion from baseline to week 12, calculated from spot urine samples using the Tanaka method. Key secondary outcomes included office blood pressure, brachial-ankle pulse wave velocity, urinary protein-to-creatinine ratio, and plasma brain natriuretic peptide. The analysis was conducted in the intention-to-treat population, using a mixed-effects model for repeated measures.

**Results:**

A total of 101 patients were randomly assigned to the intervention group (n=51) or the control group (n=50). The median (IQR) app engagement rate, calculated by dividing the number of days patients recorded blood pressure in the app by the total intervention period, was 96% (73%-99%). The mean (SD) baseline estimated glomerular filtration rate and 24-hour urinary sodium excretion were 38 (18) mL/min/1.73 m^2^ and 145 (33) mmol, respectively. A higher proportion of patients in the intervention group reported that their salt intake behaviors had “significantly improved” or “somewhat improved” by the intervention than those in the control group (35/46, 76% vs 18/47, 38%; *P*<.001). However, the mean change in estimated 24-hour urinary sodium excretion during the intervention period did not differ significantly between groups (1.4, 95% CI −12.0 to 14.7 mmol in the intervention group vs 2.5, 95% CI −10.7 to 15.6 mmol in the control group; between-group difference −1.1, 95% CI −19.8 to 17.7 mmol; *P*=.92). Secondary outcomes were not significantly different between groups. These outcomes were not altered even in a subgroup of patients reporting improved self-reported salt intake behaviors.

**Conclusions:**

The smartphone app did not reduce salt intake in patients with CKD, despite a substantial improvement in self-reported salt intake behaviors. Enhancing the intervention intensity may be necessary to effectively bridge the intention-behavior gap.

## Introduction

Excessive salt intake is a major public health concern [1], contributing to a growing number of noncommunicable diseases, such as hypertension and cardiovascular diseases, as well as mortality [2-6]. Sodium load is also detrimental to the kidney, independent of blood pressure levels [7]. Cohort studies showed an association between higher urinary sodium excretion and an elevated risk of kidney outcomes even after adjustment for blood pressure levels [8,9]. In a meta-analysis of randomized controlled trials (RCTs), short-term low-salt diets reduced albuminuria by 36% with only a modest decrease in blood pressure levels among patients with early stages of chronic kidney disease (CKD) [10]. Salt restriction may also enhance proteinuria-lowering effects of renin-angiotensin system inhibitors and improve kidney outcomes [11,12]. Thus, limiting salt intake is an essential part of dietary therapy for patients with CKD, potentially offering kidney protection beyond blood pressure control. Accordingly, the Kidney Disease Improving Global Outcomes guideline recommends a daily sodium intake of less than 2 g for patients with hypertensive CKD [13].

In real-world clinical settings, however, salt restriction is often challenging. The average urinary sodium excretion in patients with CKD has been reported to be well above the guideline recommendation (124.6-160.9 mmol/24 h [2.9-3.7 g/24  h]) [8,14-17]. Lifestyle modifications through dietary consultation and motivational interviewing guided by health care professionals have not shown consistent long-term efficacy for salt restriction [18-22]. Notably, such time-consuming individualized therapy would be costly and difficult to scale for the growing CKD population.

Smartphone app–based interventions have emerged as promising therapeutic options for hypertension. These apps aim to facilitate behavior changes and have the potential to be delivered at scale. However, RCTs examining the efficacy of these apps are scarce [23]. A recent meta-analysis showed a moderate reduction in 24-hour urinary sodium excretion (−0.39 g, 95% CI −0.50 to −0.27) by technology-supported behavior change interventions, including smartphone apps, although patients with CKD were excluded [24]. CureApp HT is a hypertension management app that provides individualized health care advice for lifestyle modification. In the HERB Digital Hypertension 1 pivotal phase III trial, a 12-week use of CureApp HT reduced blood pressure and self-reported salt intake among hypertensive patients who had not used antihypertensive medications [25]. Since the majority of participants in this trial had normal kidney function, it remains uncertain whether CureApp HT would confer similar benefits to patients with CKD who are managed by nephrologists. Here, we evaluated the efficacy of CureApp HT in patients with CKD who were treated at a tertiary referral hospital and exhibited elevated urinary sodium excretion.

## Methods

### Study Design

This was an open-label, single-center, RCT examining efficacy of CureApp HT, a smartphone app for hypertension management, on urinary sodium excretion in patients with CKD. The intervention period lasted 12 weeks, followed by a 12-week post-intervention period to assess sustainability of treatment effects. A detailed schedule is shown in Figure S1 in Multimedia Appendix 1. Patients were enrolled from the nephrology outpatient department at Osaka University Hospital, Japan, between February 2023 and November 2023. This study was conducted and reported in accordance with the CONSORT eHEALTH (Consolidated Standards of Reporting Trials of Electronic and Mobile Health Applications and Online Telehealth) checklist (Checklist 1).

### Ethical Considerations

The study protocol was approved by the University of Osaka Clinical Research Review Board (approval number 22015) and registered at Japan Registry of Clinical Trials (jRCTs052220164) on February 6, 2023. All participants provided written informed consent. Participants’ data were deidentified with unique identification codes and maintained in password-protected, access-restricted institutional databases. No financial or other compensation was provided to participants. The trial was conducted in accordance with the Declaration of Helsinki.

### Eligibility Criteria

The eligibility criteria were (1) aged 18 years or older, (2) CKD stages G1 to G5, (3) a history of hypertension (systolic blood pressure 140 mmHg or higher and/or diastolic blood pressure 90 mmHg or higher and/or use of antihypertensive medications), and (4) an estimated 24-hour urinary sodium excretion based on the Tanaka method of 100 mmol or greater [26].

Patients were excluded if (1) they were unable to handle a smartphone due to cognitive impairment or impaired finger dexterity or (2) they already used other health care smartphone apps.

### Randomization

Eligible patients were randomly assigned to the intervention group or control group in a 1:1 ratio. Randomization was performed using a computer-generated random number list with a permuted block size of 4, stratified by age (≥75 or <75 y) and estimated 24-hour urinary sodium excretion (≥170 or <170 mmol). The randomization procedure was conducted by one of the investigators who was unaware of participants’ information and had no involvement in patient enrollment.

### Interventions

Patients assigned to the intervention group used CureApp HT (CureApp Inc.), a smartphone app designed to facilitate hypertension management through lifestyle modifications and self-monitoring, for 12 weeks. This app was approved by the Pharmaceuticals and Medical Devices Agency as software as a medical device in Japan and was included in the national health insurance coverage on September 1, 2022. They also received lifestyle counseling by nephrologists as per the Clinical Practice Guideline for CKD 2018 issued by the Japanese Society of Nephrology [27]. This lifestyle counseling included general recommendations on salt restriction (targeting <6 g/d), moderation of alcohol consumption, and other standard dietary and behavioral advice. Patients in the control group received lifestyle counseling during outpatient visits, typically once every 1 to 3 months, without access to the app.

To minimize the risk of cross-contamination, all patient recruitment and follow-up were conducted individually through routine outpatient visits. No group education sessions or group-based activities were provided. Patients in both the intervention and control groups were not informed of each other’s group assignments. Furthermore, the app was accessed privately by patients in the intervention group on their personal smartphones.

Dose adjustment of antihypertensive medications, diuretics, oral antihyperglycemic medications, and potassium binders was not permitted throughout the intervention period, unless deemed clinically necessary by attending physicians.

Patients in the intervention group were instructed to download CureApp HT to their smartphones and activate it using prescription codes. They entered personal data, including age, sex, body weight, lifestyle behaviors, social background, and daily home blood pressure. These data were transferred to the cloud system, which generated individualized lifestyle modification programs.

CureApp HT is designed to enhance patients’ awareness of hypertension and facilitate lifestyle modifications and self-management through the following 3 steps:

- Step 1 (Education): Patients acquire knowledge about hypertension and lifestyle modification methods through interactive conversations with a virtual nurse within the app.
- Step 2 (Implementation): The app provides patients with personalized action plans, including salt restriction, weight loss, exercise, improvement of sleep quality, mitigation of mental stress, and reduction in alcohol consumption.
- Step 3 (Habituation): Patients set their own behavioral goals and cultivate habits with the support of the app.

Through a specialized web application, attending physicians monitored patients’ daily blood pressure levels, as entered by patients in the app, and tracked the progress of the 3 steps mentioned above.

### Primary Outcome

The primary outcome was the change in estimated 24-hour urinary sodium excretion during the intervention period, measured at baseline, week 4, and week 12. The estimated 24-hour urinary sodium excretion was calculated using the Tanaka method based on spot urine samples [26]: Estimated 24-hour urinary sodium excretion (mg) = 23 × 21.98 × {[urinary Na (mEq/L)/(urinary creatinine (mg/dL)×10)] × predicted 24-hour urinary creatinine excretion (mg/day)}^0.392^

Predicted 24-hour urinary creatinine excretion (mg/day) = [−2.04 × age (years)] + [14.89 × weight (kg)] + [16.14 × height (cm)] − 2244.45

### Secondary Outcomes

Secondary outcomes were selected to explore the potential impact of the intervention on kidney and cardiovascular risk markers, including the changes in the following parameters during the intervention period, measured at baseline and week 12: (1) office blood pressure, (2) brachial-ankle pulse wave velocity (baPWV), (3) urinary protein-to-creatinine ratio (UPCR), (4) urinary potassium-to-creatinine ratio, (5) urinary sodium-to-potassium ratio, (6) estimated glomerular filtration rate (eGFR), (7) brain natriuretic peptide (BNP), (8) body weight, and (9) the number of antihypertensive medications.

Office blood pressure was measured using an automated oscillometric blood pressure monitor (Omron HEM-907). Patients self-measured their blood pressure after resting quietly in a seated position for 5 minutes without attendance of medical personnel. The average value of 2 measurements at 1-minute intervals was used for statistical analysis.

The baPWV was measured using a noninvasive automated device (Omron BP-203RPEIII) after patients rested for 5 minutes in a supine position. This device measured baPWV twice simultaneously on the right and left sides. The average value of these 4 measurements was used for statistical analysis.

eGFR was calculated using the Japanese standard formula: 194 × creatinine^-1.094^ × age^-0.287^ (if female, × 0.739) [28].

### Questionnaire Survey on Salt Intake Behaviors

After the 12-week postintervention period, patients were asked to assess how much their lifestyle behaviors had improved by responding to the following question: *“*To what extent has your lifestyle related to (1) salt intake, (2) exercise, (3) sleep quality, (4) mental stress, and (5) alcohol consumption improved as a result of participating in this trial?.” Patients selected one of the following options: “significantly improved,” “somewhat improved,” “not much improved,” or “not improved at all.” Regarding research objectives, only the salt-intake item was included in a between-group comparison in the present analysis.

### Statistical Analysis

A previous observational study reported that every 40 mmol/24 h increase in urinary sodium excretion was associated with a 10% higher hazard of cardiovascular events in patients with CKD [9]. Based on this finding, we considered a 40 mmol/24 h decrease in urinary sodium excretion to be clinically relevant. According to the 2-arm pilot trial sample size calculation [29], 96 patients (48 in each group) were required to provide 90% power, with a 2-sided type I error level of .05, to detect a between-group difference of 40 mmol/24 h with a SD of 60 mmol/24 h. To account for a 5% dropout rate, we planned to recruit 100 patients.

Between-group comparisons were conducted using the Student *t* test or Mann-Whitney test for continuous variables and the chi-square test or Fisher exact test for categorical variables, as appropriate.

The primary analysis was conducted in the intention-to-treat population, defined as all randomized patients who used CureApp HT for at least one day or received at least one lifestyle counseling session. We employed a mixed-effects model for repeated measures (MMRM) with an unstructured covariance matrix to compare between-group differences in the primary and secondary outcomes. Fixed effects in the MMRM were treatment groups, categoric time, treatment-by-time interaction, and stratification factors (age and baseline estimated 24-h urinary sodium excretion). No multiplicity adjustments for each outcome were made in this proof-of-concept study. There were no missing data on baseline estimated 24-hour urinary sodium excretion. The missing data after baseline were not imputed but were instead handled directly within the MMRM.

Prespecified subgroup analyses were performed based on baseline age (below or above the median), baseline estimated 24-hour urinary sodium excretion (below or above the median), and app engagement rates calculated by dividing the number of days patients recorded blood pressure in the app by the total intervention period (≥71.4% or <71.4%). Effect modifications were tested by incorporating treatment-by-subgroup interactions into the MMRM.

Sensitivity analyses were performed after excluding patients (1) who did not reach step 3 and (2) whose family members assisted with app usage due to patients’ difficulty in operating a smartphone.

An exploratory analysis was conducted in which patients were categorized into 4 groups using the results of the questionnaire survey on salt intake behaviors (“not improved at all,” “not much improved,” “somewhat improved,” and “significantly improved”). A linear regression model was used to evaluate the association between the self-reported behavior category and the change in estimated 24-hour urinary sodium excretion.

All statistical analyses were performed using the Stata/IC software (version 18.0; Stata Corp). A *P* value of <.05 was considered statistically significant.

## Results

### Patient Characteristics

**Figure 1.**
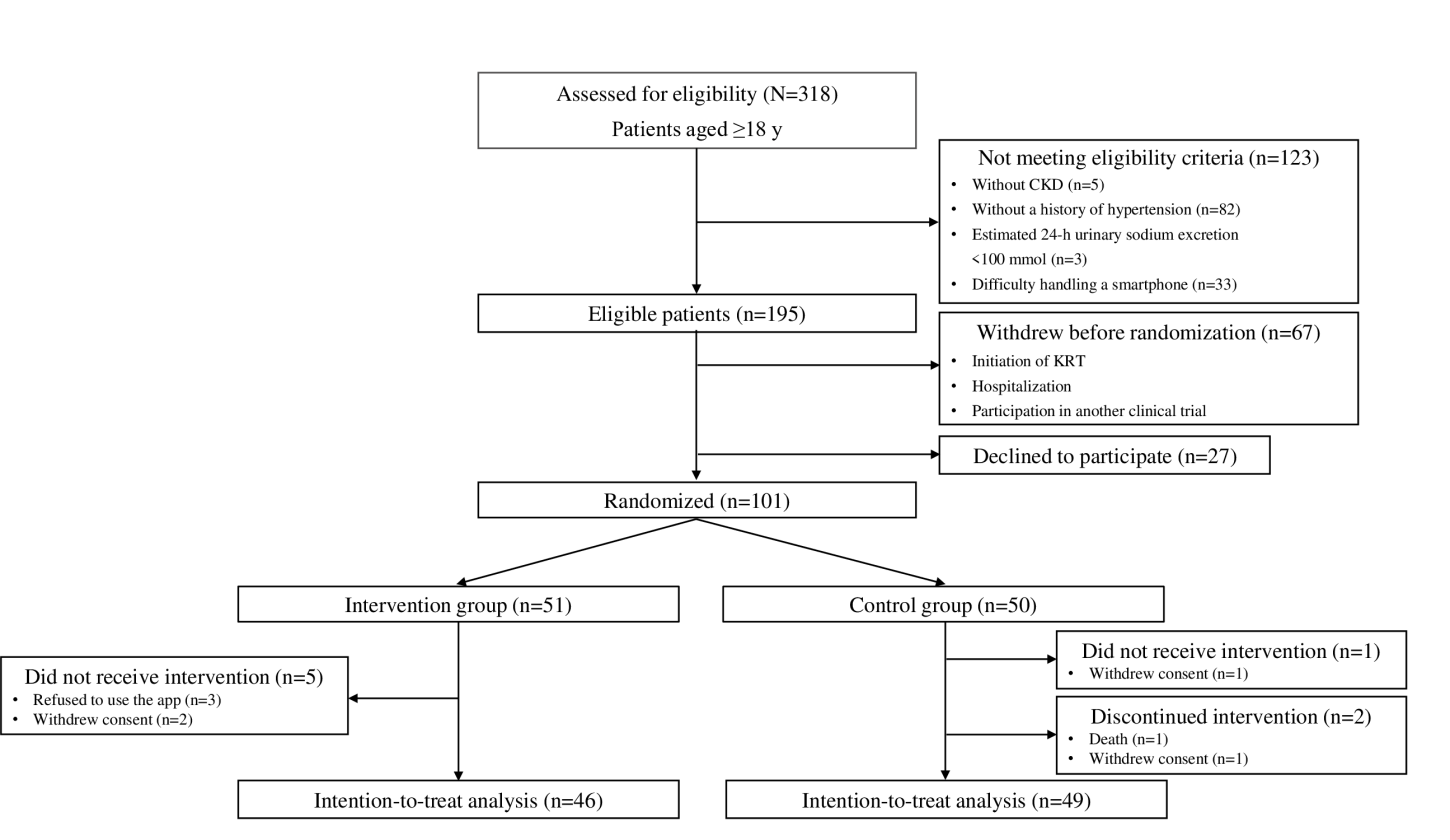
Flow diagram of the study. CKD: chronic kidney disease; KRT: kidney replacement therapy.

A total of 101 patients were randomly assigned to the intervention group (n=51) or the control group (n=50) (Figure 1). In the intervention group, 3 patients refused to use the app and 2 patients withdrew consent before using the app, while no patients dropped out during the intervention period. In the control group, 1 patient withdrew consent before the intervention period, and 2 patients dropped out during the intervention period.

The baseline characteristics are shown in Table 1. The mean (SD) age, blood pressure, and eGFR were 66 (14) years, 131 (19)/79 (14) mmHg, and 38 (18) mL/min/1.73 m^2^, respectively. Patients in the intervention group showed slightly lower systolic blood pressure and eGFR than those in the control group. The estimated 24-hour urinary sodium excretion at baseline was 145 (33) mmol and was well-balanced between groups.

**Table 1.** Baseline characteristics of the study patients.

Characteristics	Total (N=101)	Intervention group (n=51)	Control group (n=50)	*P* value^a^
Age, years, mean (SD)	66 (14)	67 (14)	66 (14)	.56
Male, n (%)	63 (62)	29 (57)	34 (68)	.25
BMI, kg/m^2^, mean (SD)	23.9 (3.6)	23.9 (3.7)	23.9 (3.5)	.98
Systolic BP^b^, mmHg, mean (SD)	131 (19)	128 (20)	134 (16)	.10
Diastolic BP, mmHg, mean (SD)	79 (14)	79 (15)	79 (14)	.76
Diabetes mellitus, n (%)	36 (36)	15 (29)	21 (42)	.19
Cardiovascular comorbidities, n (%)	20 (20)	9 (18)	11 (22)	.58
CKD^c^ stages, n (%)				.14
G2	12 (12)	5 (10)	7 (14)	
G3	53 (52)	26 (51)	27 (54)	
G4	28 (28)	14 (27)	14 (28)	
G5	8 (8)	6 (12)	2 (4)	
Smoking history, n (%)				.33
Never smoked	52 (51)	30 (59)	22 (44)	
Former smoker	40 (40)	17 (33)	23 (46)	
Current smoker	9 (9)	4 (8)	5 (10)	
Hemoglobin, g/dL, mean (SD)	12.9 (1.8)	12.7 (1.7)	13.1 (1.9)	.22
Albumin, g/dL, mean (SD)	4 (0.4)	4 (0.3)	4.1 (0.4)	.43
Potassium, mEq/L, mean (SD)	4.5 (0.5)	4.5 (0.6)	4.5 (0.5)	.88
Creatinine, mg/dL, mean (SD)	1.7 (0.9)	1.8 (1.1)	1.5 (0.6)	.09
eGFR^d^, mL/min/1.73 m^2^, mean (SD)	38 (18)	35 (18)	40 (18)	.18
Calcium, mg/dL, mean (SD)	9.4 (0.4)	9.5 (0.5)	9.4 (0.4)	.63
Phosphate, mg/dL, mean (SD)	3.5 (0.7)	3.6 (0.6)	3.4 (0.7)	.20
CRP^e^, mg/dL, median (IQR)	0.1 (0.0-0.2)	0.1 (0.0-0.2)	0.1 (0.0-0.1)	.62
UPCR^f^, g/gCre, median (IQR)	0.28 (0.10-0.74)	0.28 (0.09-0.97)	0.27 (0.10-0.53)	.62
BNP^g^, pg/mL, median (IQR)	28 (11-63)	27 (9-43)	34 (15-69)	.29
Estimated 24-h urinary sodium excretion, mmol, mean (SD)	145 (33)	143 (29)	147 (37)	.56

a *P* values indicate differences between intervention and control groups.

b BP: blood pressure.

c CKD: chronic kidney disease.

d eGFR: estimated glomerular filtration rate.

e CRP: C-reactive protein.

f UPCR: urinary protein-to-creatinine ratio.

g BNP: B-type natriuretic peptide.

### Questionnaire Survey on Salt Intake Behaviors

**Figure 2.**
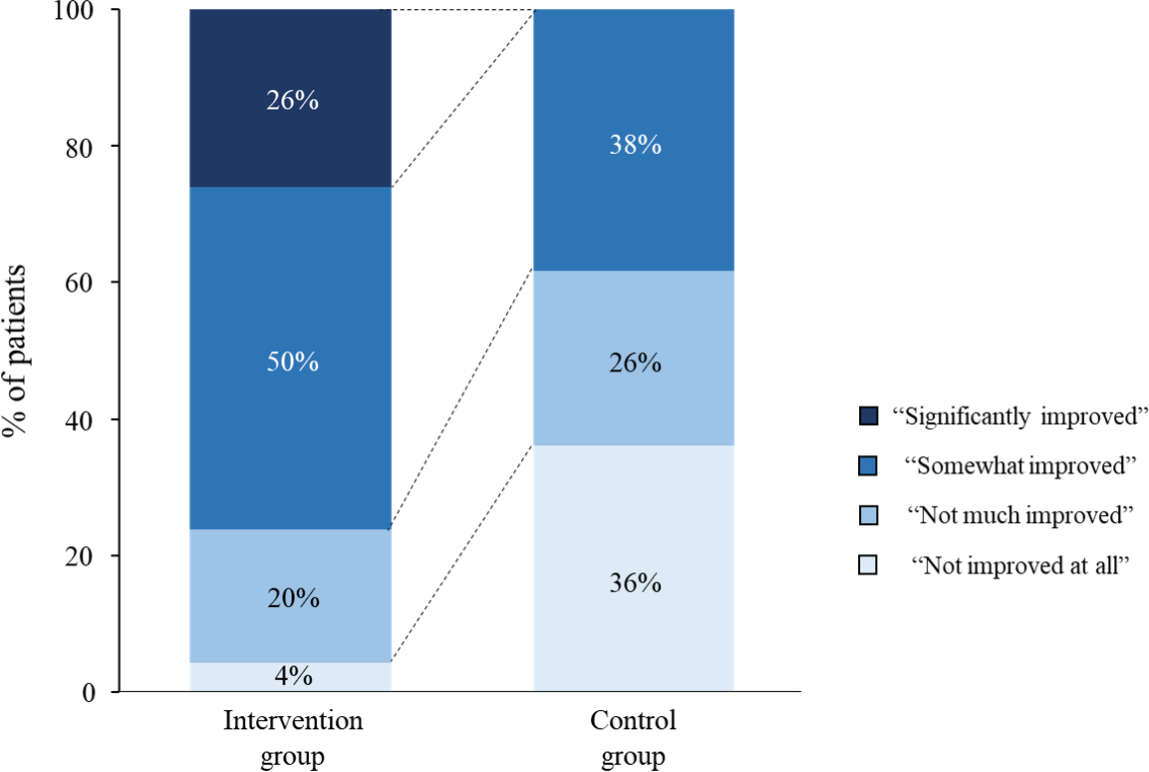
Questionnaire survey on salt intake behaviors. Self-reported salt intake behaviors were significantly improved in the intervention group compared with the control group. Patients were asked, "To what extent has your lifestyle related to salt intake improved as a result of participating in this trial?" In the intervention group, 35 of 46 (76%) patients reported that their salt intake behavior had “significantly improved” or “somewhat improved” compared with 18 of 47 (38%) in the control group (*P*<.001; *χ*^2^_3_=24.87).

In the intervention group, 35 of 46 (76%) patients reported that their salt intake behavior had *“*significantly improved*”* or “somewhat improved” by the intervention, compared with 18 of 47 (38%) in the control group (*χ*^2^_3_=24.87; *P*<.001; Figure 2, Table S1 in Multimedia Appendix 1).

### Primary Outcome

**Figure 3.**
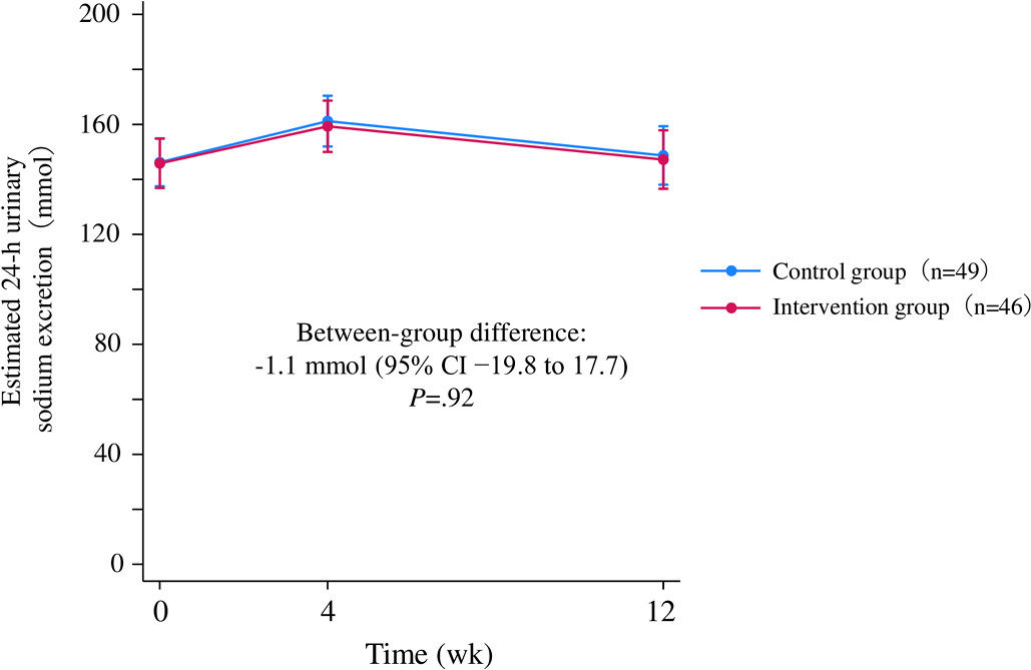
Estimated 24-hour urinary sodium excretion during the intervention period. Data are shown for the intention-to-treat population (46 patients in the intervention group and 49 patients in the control group). A mixed-effects model for repeated measures with an unstructured covariance matrix was used to compare between-group changes. The model included age, baseline estimated 24-hour urinary sodium excretion, randomized groups, time, and interaction between randomized groups and time as fixed effects, and subjects as a random effect. Error bars represent 95% CIs.

The mean change in estimated 24-hour urinary sodium excretion from baseline to week 12 was 1.4 (95% CI −12.0 to 14.7) mmol in the intervention group and 2.5 (95% CI −10.7 to 15.6) mmol in the control group, with no significant between-group difference (−1.1 mmol, 95% CI −19.8 to 17.7; *P*=.92) (Figure 3, Table 2). Age, baseline estimated 24-hour urinary sodium excretion, and app engagement rates did not significantly modify the results (Table S2 in Multimedia Appendix 1).

After the 12-week postintervention period, the mean change in the estimated 24-hour urinary sodium excretion from baseline was not significantly different between the groups; 4.7 (95% CI −8.9 to 18.4) and 13.9 (95% CI 0.4-27.3) mmol in the intervention and control groups, respectively (between-group difference, −9.1 mmol, 95% CI −28.3 to 10.0; *P*=.35).

**Table 2.** Summary of the primary and secondary outcomes.^a^

Outcome	Intervention group (n=46)			Control group (n=49)			Difference-in-changes^b^ (95% CI)	*P* value^c^
	Baseline, mean (95% CI)	Week 12, mean (95% CI)	Within-group change^d^, mean (95% CI)	Baseline, mean (95% CI)	Week 12, mean (95% CI)	Within-group change^d^, mean (95% CI)		
Primary outcome								
Estimated 24-h urinary sodium excretion, mmol	145.8 (136.8 to 154.8)	147.2 (136.6 to 157.9)	1.4 (−12 to 14.7)	146.2 (137.5 to 154.9)	148.7 (138.1 to 159.3)	2.5 (−10.7 to 15.6)	−1.1 (−19.8 to 17.7)	.92
Secondary outcomes								
Office SBP^e^, mmHg	129 (125 to 133)	130 (126 to 134)	1 (−4 to 6)	131 (127 to 135)	129 (126 to 133)	−2 (−7 to 3)	3 (−4 to 10)	.43
Office DBP^f^, mmHg	79 (77 to 81)	78 (76 to 80)	−1 (-4 to 2)	79 (77 to 81)	77 (75 to 80)	−2 (−5 to 1)	1 (−4 to 5)	.72
Mean baPWV^g^, cm/s	1687 (1648 to 1726)	1708 (1668 to 1748)	21 (−35 to 76)	1697 (1659 to 1735)	1726 (1687 to 1765)	30 (−25 to 84)	−9 (−87 to 69)	.82
UPCR^h^, g/gCre	0.75 (0.63 to 0.87)	0.79 (0.66 to 0.91)	0.04 (−0.14 to 0.21)	0.74 (0.62 to 0.86)	0.91 (0.78 to 1.03)	0.16 (−0.01 to 0.33)	−0.13 (−0.37 to 0.12)	.31
uK/uCre^i^, mmol/gCre	0.46 (0.41 to 0.52)	0.41 (0.35 to 0.46)	−0.06 (−0.14 to 0.02)	0.44 (0.38 to 0.49)	0.46 (0.40 to 0.51)	0.02 (−0.06 to 0.10)	−0.08 (−0.19 to 0.03)	.18
uNa/uK^j^	3.22 (2.87 to 3.57)	3.30 (2.95 to 3.65)	0.08 (−0.42 to 0.57)	3.27 (2.93 to 3.61)	3.11 (2.75 to 3.46)	−0.16 (−0.65 to 0.33)	0.24 (−0.46 to 0.94)	.50
eGFR^k^, mL/min/1.73 m^2^	38 (37 to 39)	38 (37 to 39)	0 (−1.4 to 1.5)	38 (37.1 to 39)	39.3 (38.3 to 40.4)	1.3 (−0.1 to 2.7)	−1.3 (-3.3 to 0.8)	.22
BNP^l^, pg/mL	54.2 (42 to 66.4)	32.1 (19.9 to 44.3)	−22.1 (−39.3 to −4.8)	56.8 (45 to 68.6)	49.8 (37.6 to 62)	−7 (−24 to 10)	−15.1 (−39.3 to 9.1)	.22
Body weight, kg	64.1 (63.8 to 64.5)	64.4 (64.1 to 64.8)	0.3 (−0.2 to 0.8)	64.1 (63.8 to 64.5)	64.3 (63.9 to 64.6)	0.2 (−0.3 to 0.7)	0.2 (−0.6 to 0.9)	.66
The number of antihypertensive medications	1.22 (1.19 to 1.26)	1.25 (1.21 to 1.29)	0.03 (−0.02 to 0.08)	1.22 (1.19 to 1.26)	1.22 (1.19 to 1.26)	0 (−0.05 to 0.05)	0.03 (−0.05 to 0.10)	.46

a Results were obtained from the mixed-effects models for repeated measures (MMRM) with an unstructured covariance matrix. Fixed effects in the MMRM were treatment group, categoric time, the treatment-by-time interaction, age, and baseline values of each outcome variable. Data presented as predicted mean (95% CI).

b Between-group differences in the changes from baseline to week 12. Positive values indicate that the intervention group exhibited greater increases than the control group.

c *P* values for difference-in-changes.

d Positive values indicate an increase from baseline to week 12.

e SBP: systolic blood pressure.

f DBP: diastolic blood pressure.

g baPWV: brachial-ankle pulse wave velocity.

h UPCR: urinary protein-to-creatinine ratio.

i uK/uCre: urinary potassium-to-creatinine ratio.

j uNa/uK: urinary sodium-to-potassium ratio.

k eGFR: estimated glomerular filtration rate.

l BNP: B-type natriuretic peptide.

### Secondary Outcomes

There were no significant between-group differences in the changes in blood pressure, baPWV, UPCR, urinary potassium-to-creatinine ratio, urinary sodium-to-potassium ratio, eGFR, BNP, body weight, and the number of antihypertensive medications during the intervention period (Table 2, Figure S2 in Multimedia Appendix 1). No adverse events related to app use were reported.

The median (IQR) app engagement rate was 96% (73%-99%). At the end of the intervention period, 35 of 46 (76%) patients who used the app at least once completed Step 1, and 22 of 46 (48%) completed both Step 1 and Step 2.

### Sensitivity Analyses

The results were not substantially altered after excluding patients who did not reach Step 3 (24/46, 52%) or whose family members helped use the app (1/46, 2%) (Table S3 in Multimedia Appendix 1).

### Exploratory Analysis

Participants were categorized into 4 groups according to the questionnaire survey on salt intake behaviors at week 12. There were no significant linear trends between self-reported salt intake behavior and the change in urinary sodium excretion in either the intervention or control group (Table S4 in Multimedia Appendix 1).

## Discussion

### Overview

In this single-center, open-label, RCT involving patients with CKD, we observed a substantial improvement in self-reported salt intake behaviors after 12 weeks of CureApp HT use. However, the app did not improve the primary outcome, estimated 24-hour urinary sodium excretion, a surrogate measure of actual salt intake. Secondary outcomes, including blood pressure, baPWV, UPCR, and BNP, also did not differ significantly between groups. Notably, these outcomes did not improve even in a subgroup of patients who reported significant improvements in salt intake behaviors, suggesting a gap between self-awareness and actual behaviors regarding dietary salt intake. Bridging this gap will be a major challenge that digital therapeutics need to address.

### Principal Findings

Salt restriction is essential in the management of CKD to achieve optimal blood pressure control. Associations between urinary sodium excretion and kidney outcomes have been reported to be independent of blood pressure levels [8,9]. This suggests that mechanisms beyond blood pressure elevation may contribute to salt-induced kidney damage. Basic studies have shown that a high-salt diet accelerates kidney damage through several mechanisms, including Rac1-mediated mineralocorticoid receptor activation [7], inhibition of tissue remodeling by macula densa cells [30], and inflammation and fibrosis induced by Y-box binding protein-1 [31]. These preclinical findings imply that salt restriction may confer kidney protection even when blood pressure levels are normal. Although this concept has not been clinically tested, short-term low-salt diets significantly reduced albuminuria with only modest impacts on blood pressure levels [10,11]. Additionally, salt restriction may enhance the kidney-protective effects of renin–angiotensin system inhibitor [12]. Therefore, although our patients had well-controlled blood pressure levels, salt restriction may still be beneficial in reducing residual salt toxicity to the kidney. Thus, urinary sodium excretion is a clinically relevant outcome in this trial.

We identified a substantial discrepancy between patients’ self-reported salt restriction behaviors and objectively measured urinary sodium excretion. A similar finding was reported in the HERB Digital Hypertension 1 trial, in which CureApp HT significantly improved self-reported salt intake but had minimal impact on the urinary sodium-to-creatinine ratio [25]. These findings suggest that intentions do not necessarily translate into behavioral changes, highlighting the presence of an intention-behavior gap. Psychological behavior theories, such as the Theory of Planned Behavior and Self-Determination Theory, assume that intention is the proximal determinant of behavior. However, a “gap” exists between intention and behavior [32]. There is only a modest correlation between intentions and actual behaviors [33,34]; intention accounts for only 27% of the variance in dietary habits [35], and individuals often underestimate their salt intake [36,37]. This gap is influenced by several moderators, including (1) social environment, (2) behavioral inertia, and (3) sodium palatability. The social environment plays a significant role in determining salt intake behaviors. Even when patients recognize the importance of salt restriction, it is not feasible in situations where access to fresh, low-sodium foods is limited and processed foods (including prepackaged and fast food) are prevalent. In Japan, many traditional foods, such as soy sauce and pickled vegetables, are high in salt. These social and environmental factors may impede patients’ intention to adhere to a low-salt diet despite their increased awareness of salt restriction. Public health policies and social support must be implemented, in collaboration with multiple stakeholders such as the food industry, to create an environment where low-salt foods are more readily accessible [38].

Behavioral inertia is another barrier that exacerbates the intention-behavior gap. Established eating habits are a putative determinant of human salt intake [39]. A prior study found no association between the intention to change salt intake habits and the actual purchasing behavior of low-salt foods [40]. Because our patients were older, they were likely to maintain long-established dietary and cooking habits, which are inherently resistant to change, although age did not significantly modify the results in subgroup analysis. Regular face-to-face care from nephrologists may have led patients to assume that their current salt intake was already acceptable, even though their salt intake was beyond the target range. This false perception may reinforce patients’ psychological resistance to behavioral modifications for salt restriction proposed by the app. Importantly, health benefits from salt restriction are not always immediately apparent. The delayed rewards of long-term health benefits obtained from salt restriction, in exchange for immediate gratification of enjoying salty foods, further complicate maintaining salt restriction as a regular habit [40].

Finally, sodium palatability may also contribute to the intention-behavior gap among patients with CKD. Under sodium-sated conditions, humans exhibit an attraction toward low concentrations of sodium but an aversion toward high concentrations of sodium [41]. However, patients with CKD typically have a higher threshold for salty taste perception than healthy individuals [42,43], and their aversive response to high sodium concentrations may be diminished [44]. Such an altered salty taste perception, coupled with an increased tolerance for excess salt, likely enhances sodium palatability of these patients, making dietary salt restriction even more challenging.

To bridge the intention-behavior gap regarding salt restriction, it may be necessary to optimize the intensity of intervention. Greaves et al [45] reported that intervention intensity, such as the number of sessions and total contact time, was related to self-reported dietary changes. For example, in the Trials of Hypertension Prevention, which demonstrated substantial efficacy of comprehensive education and counseling on salt restriction, intensive and meticulous interventions were conducted (8 group counseling sessions and 2 individual sessions over the first 3 mo, followed by periodic group meetings and individual telephone or in-person contacts throughout the follow-up) [46]. In patients with CKD, theory-based behavioral interventions, using both face-to-face and web-based approach (e-coaching and group meetings), have shown only modest and temporary effects on salt restriction, potentially due to a relatively low intensity of these interventions [20,21]. The intensity of the smartphone-based digital intervention might be even weaker, particularly for patients with CKD who likely have a large intention-behavior gap, owing to the absence of human involvement. Therefore, enhancing intervention intensity in smartphone-based approaches, potentially by incorporating more interactive and individualized elements, may be essential to improve their effectiveness in promoting sustained salt restriction.

### Limitations

This trial has several limitations, one of which is the open-label design. However, blinding was impractical given the nature of the intervention. Since the measurement of urinary sodium excretion was objective, potential bias from the open-label design was likely mitigated. Second, the sample size was modest and the trial duration was relatively short. However, given the neutral result of the primary outcome, it is unlikely that a larger and longer-term trial would have produced a positive result. Third, although the accuracy of the Tanaka method for estimating 24-hour urinary sodium excretion from a spot urine test has been validated in Japanese patients with CKD [47], its accuracy may still be inferior to that of a 24-hour urine collection. However, if dietary salt intake had been meaningfully reduced, secondary outcomes, including blood pressure, UPCR, and BNP, would likely have shown improvement. Fourth, the questionnaire survey on salt intake behaviors may have been subject to social-desirability bias, potentially leading to an overestimation of the proportion of patients reporting improvements. However, the substantial difference between the intervention and control groups suggests that the app did have some impact at least on patients’ intention. Fifth, the study did not assess the potential time lag between knowledge acquisition and actual behavioral change, which may have contributed to the limited correspondence between self-reported behavior and objective outcomes. Finally, since our study included patients who were willing to use the app, the generalizability of our findings to the broader real-world population, particularly outside of Japan, is uncertain.

### Conclusions

CureApp HT, a hypertension management app that provides individualized advice and self-monitoring for lifestyle modifications and salt restriction, was not effective in reducing urinary sodium excretion among patients with CKD, despite substantial improvement in self-reported salt intake behaviors. Secondary outcomes, including blood pressure, baPWV, UPCR, and BNP, also did not differ significantly between groups, further indicating that the app did not improve other cardiovascular and kidney risk markers. These results should be interpreted with caution due to the open-label design and the relatively short duration of the trial. Nonetheless, these findings suggest the presence of an intention-behavior gap for salt restriction, which might be addressed by enhancing the intensity of smartphone app–based interventions.

## Supplementary material

10.2196/68447 Multimedia Appendix 1 Questionnaire results on salt intake, subgroup and sensitivity analyses, and study design.

10.2196/68447 Checklist 1 CONSORT eHEALTH checklist (version 1.6.1).

## Abbreviations

baPWV: brachial-ankle pulse wave velocity
BNP: brain natriuretic peptide
CKD: chronic kidney disease
CONSORT eHEALTH: Consolidated Standards of Reporting Trials of Electronic and Mobile Health Applications and Online Telehealth
eGFR: estimated glomerular filtration rate
MMRM: mixed-effects model for repeated measures
RCT: randomized controlled trial
UPCR: urinary protein-to-creatinine ratio
