# Supplementary material for: Effects of a Hypertension Management Mobile App on Urinary Sodium Excretion in Patients With Chronic Kidney Disease: Randomized Controlled Trial
Source: JMIR Mhealth Uhealth. 2026 Mar 16;14:e68447. doi: 10.2196/68447 (PMC12991191; doi:10.2196/68447)
Supplement: Multimedia Appendix 1 [file mhealth-v14-e68447-s001.docx]

**Supplemental Materials**

**Effects of a Hypertension-Management Mobile App on Urinary Sodium Excretion in Patients with CKD: A Randomized Controlled Trial**

**Supplemental Tables**

**Supplemental Table 1. Questionnaire survey on salt intake behaviors**

**Supplemental Table 2. Subgroup analyses for the changes in estimated 24-hour urinary sodium excretion**

**Supplemental Table 3. Sensitivity analyses for the changes in estimated 24-hour urinary sodium excretion**

**Supplemental Table 4. Exploratory analysis for the changes in estimated 24-hour urinary sodium excretion**

**Supplemental Figures**

**Supplemental Figure 1. Study design**

**Supplemental Figure 2. Results for secondary outcomes**

**Supplemental Table 1. Questionnaire survey on salt intake behaviors**

|  | Response to the questionnaire survey, number (%) | | | |  |
| --- | --- | --- | --- | --- | --- |
|  | “Significantly improved” | “Somewhat improved” | “Not much improved” | “Not improved at all” | Total |
| Intervention Group | 12 (26%) | 23 (50%) | 9 (20%) | 2 (4%) | 46 |
| Control Group | 0 (0%) | 18 (38%) | 12 (26%) | 17 (36%) | 47 |

Data are expressed as numbers (%).

In the control group, while 49 patients were included in the intention-to-treat analysis, 2 patients dropped out during the intervention period. Consequently, 47 patients responded to this questionnaire survey.

**Supplemental Table 2. Subgroup analyses for the changes in estimated 24-hour urinary sodium excretion**

|  | | Intervention group | Control group | Difference-in-changes^a^  [95% confidence interval] | *P* value^b^ | *P* for interaction |
| --- | --- | --- | --- | --- | --- | --- |
| Age | ≥68 years | n=22 | n=27 | -12.0 [-36.5 to 12.5] | 0.34 | 0.24 |
|  | <68 years | n=24 | n=22 | 10.7 [-18.0 to 39.3] | 0.47 |  |
| Baseline estimated 24-hour urinary sodium excretion | ≥141.1 mmol | n=25 | n=23 | 2.5 [-20.6 to 25.5] | 0.79 | 0.73 |
|  | <141.1 mmol | n=21 | n=26 | -4.0 [-31.5 to 23.6] | 0.78 |  |
| App engagement rate | ≥71.4 % | n=35 | n=49 | -4.1 [-24.6 to 16.3] | 0.69 | 0.40 |
|  | <71.4 % | n=11 | n=49 | 9.2 [-23.0 to 41.4] | 0.57 |  |

^a^Between-group differences in the changes from baseline to week 12. Positive values indicate that the intervention group exhibited greater increases than the control group.

^b^*P* values for difference-in-changes.

The app engagement rate was calculated by dividing the number of days on which patients recorded blood pressure data in the app by the total number of days during the intervention period.

**Supplemental Table 3. Sensitivity analyses for the changes in estimated 24-hour urinary sodium excretion**

|  | Intervention group ((1) n=22, (2) n=45) | | | Control group (n=49) | | |  |  |
| --- | --- | --- | --- | --- | --- | --- | --- | --- |
|  | Baseline | Week 12 | Within-group change^a^ | Baseline | Week 12 | Within-group change^a^ |  |  |
| ***1) Excluding patients who did not reach Step 3*** | Mean [95%CI] | Mean [95%CI] | Mean [95% CI] | Mean [95%CI] | Mean [95%CI] | Mean [95% CI] | Difference-in-changes^b^  [95% CI] | *P*-value^c^ |
| Estimated 24-hour urinary sodium excretion, mmol | 143.8  [130.0 to 157.6] | 139.9  [125.1 to 154.7] | -3.9  [-23.7 to 16.0] | 146.1  [136.9 to 155.4] | 148.5  [138.3 to 158.7] | 2.4  [-11.1 to 15.9] | -6.3  [-30.3 to 17.7] | 0.61 |
| ***2)***  ***Excluding patients whose family members helped use the app*** | Mean [95%CI] | Mean [95%CI] | Mean [95% CI] | Mean [95%CI] | Mean [95%CI] | Mean [95% CI] | Difference-in-changes^b^  [95% CI] | *P*-value^c^ |
| Estimated 24-hour urinary sodium excretion, mmol | 145.5  [136.3 to 154.6] | 146.5  [135.7 to 157.3] | 1.0  [-12.5 to 14.6] | 146.1  [137.3 to 154.8] | 148.5  [137.9 to 159.2] | 2.5  [-10.8 to 15.7] | -1.4  [-20.4 to 17.5] | 0.88 |

Results were obtained from the mixed-effects models for repeated measures (MMRM) with an unstructured covariance matrix. Fixed effects in the MMRM were treatment group, categoric time, the treatment-by-time interaction, age and estimated 24-hour urinary sodium excretion at baseline. Data presented as predicted mean [95% confidence interval].

^a^Positive values indicate an increase from baseline to week 12.

^b^Between-group differences in the changes from baseline to week 12. Positive values indicate that the intervention group exhibited greater increases from baseline to week12 than the control group.

^c^*P* values for difference-in-changes.

Abbreviation: CI, confidence interval.

**Supplemental Table 4. Exploratory analysis for the changes in estimated 24-hour urinary sodium excretion**

|  |  | Response to the questionnaire survey | | | |  |
| --- | --- | --- | --- | --- | --- | --- |
|  |  | “Significantly improved” | “Somewhat improved” | “Not much improved” | “Not improved at all” | P for trend |
| Intervention Group | n | 12 | 23 | 9 | 2 |  |
|  | Within-group change^a^ | -3.9 [-23.1 to 15.3] | 6.7 [-12.6 to 26.0] | 3.5 [-19.6 to 26.6] | -38.3 [-58.1 to -18.5] | 0.69 |
| Control Group | n | 0 | 18 | 12 | 17 |  |
|  | Within-group change^a^ | 0 | 2.3 [-18.4 to 22.9] | -9.8 [-36.4 to 16.8] | 10.0 [-16.6 to 36.7] | 0.52 |

Results were obtained from mixed-effects models for repeated measures (MMRM) with an unstructured covariance matrix. Fixed effects in the MMRM were treatment group, categoric time, the treatment-by-time interaction, age and estimated 24-hour urinary sodium excretion at baseline. Data presented as predicted mean [95% confidence interval].

^a^Positive values indicate an increase from baseline to week 12.

**Supplemental Figure 1. Study design**

**
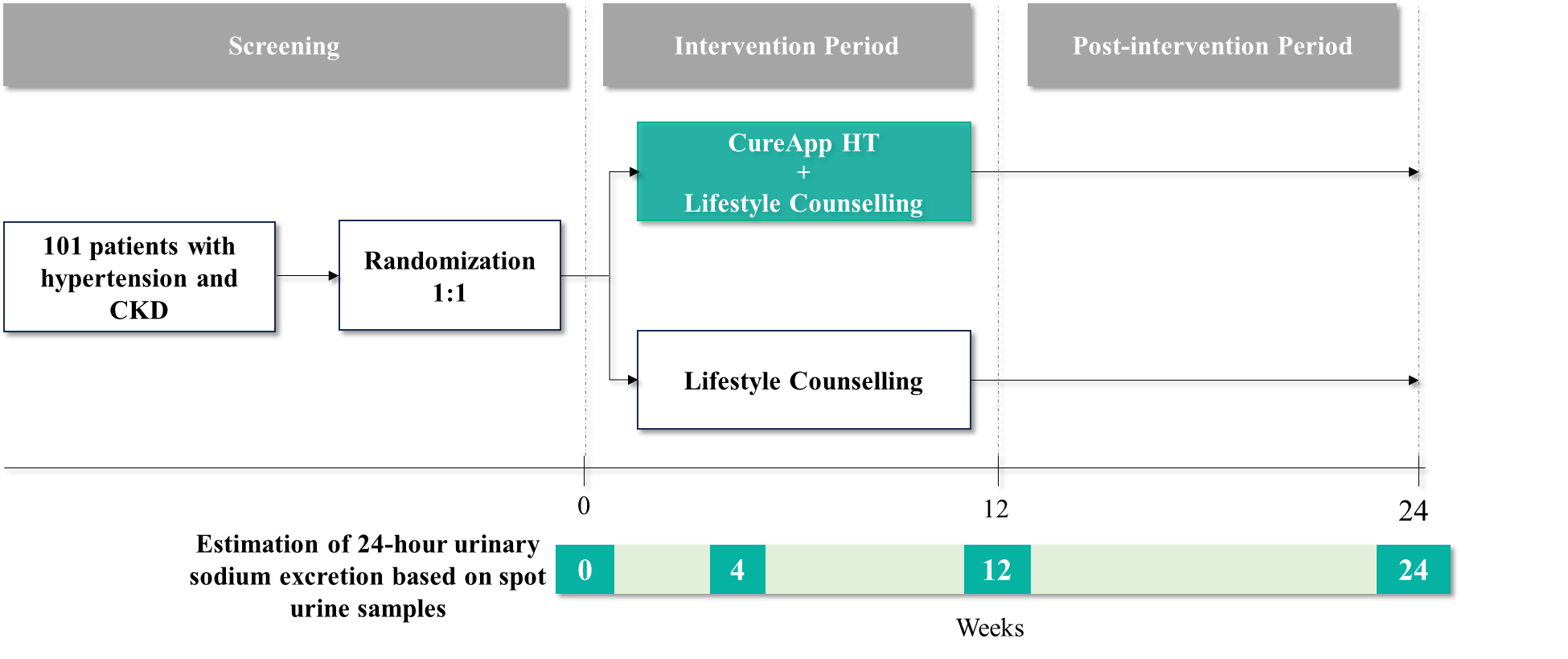
**

101 patients with hypertension and CKD were randomized to the intervention group (use of CureApp HT plus lifestyle counselling) and control group (lifestyle counselling only) in a 1:1 ratio. The intervention period was 12 weeks, followed by a 12-week post-intervention period. The primary outcome, estimated 24-hour urinary sodium excretion, was assessed at week 0, 4, and 12.

Abbreviation: CKD, chronic kidney disease.

**Supplemental Figure 2. Results for secondary outcomes**

**
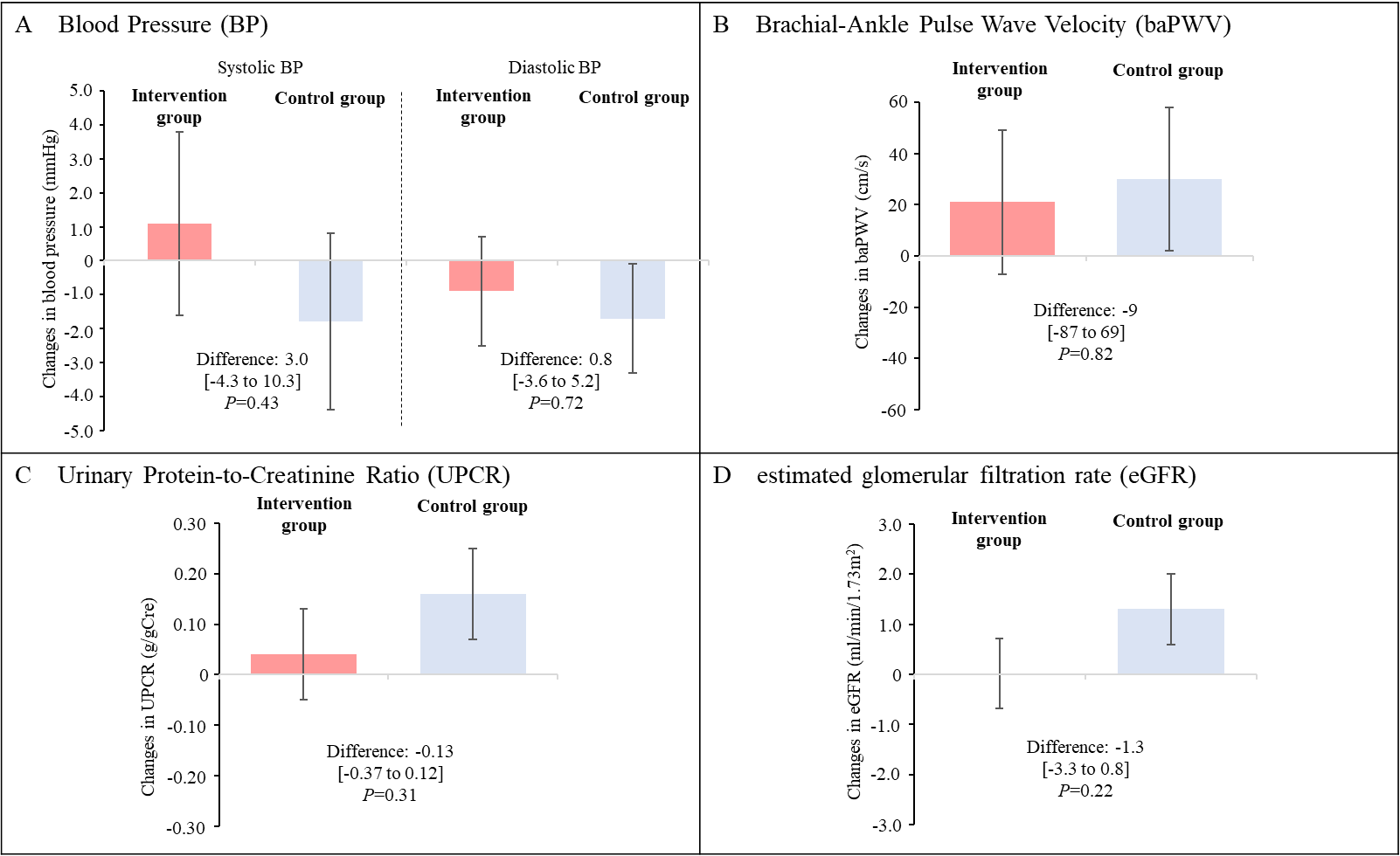
**

Shown are data for the intention-to-treat population (46 patients in the intervention group and 49 patients in the control group). A mixed-effects model for repeated measures (MMRM) with an unstructured covariance matrix was used to compare between-group changes. The model included age, baseline values for each variable, randomized groups, time, and interactions between randomized groups and time as fixed effects, and subjects as a random effect. Bars indicate changes from baseline to week 12 within each group. Positive values indicate increase from baseline to week 12.
